# Utilization of, Perceptions on, and Intention to Use AI Chatbots Among Medical Students in China: National Cross-Sectional Study

**DOI:** 10.2196/57132

**Published:** 2024-10-28

**Authors:** Wenjuan Tao, Jinming Yang, Xing Qu

**Affiliations:** 1 Institute of Hospital Management, West China Hospital, Sichuan University, No. 37, Guoxue Xiang, Chengdu, 610041, China, 86 13880713452; 2 West China Biomedical Big Data Center, West China Hospital, Sichuan University, Chengdu, China; 3 Med-X Center for Informatics, Sichuan University, Chengdu, China

**Keywords:** medical education, artificial intelligence, UTAUT model, utilization, medical students, cross-sectional study, AI chatbots, China, acceptance, electronic survey, social media, medical information, risk, training, support

## Abstract

**Background:**

Artificial intelligence (AI) chatbots are poised to have a profound impact on medical education. Medical students, as early adopters of technology and future health care providers, play a crucial role in shaping the future of health care. However, little is known about the utilization of, perceptions on, and intention to use AI chatbots among medical students in China.

**Objective:**

This study aims to explore the utilization of, perceptions on, and intention to use generative AI chatbots among medical students in China, using the Unified Theory of Acceptance and Use of Technology (UTAUT) framework. By conducting a national cross-sectional survey, we sought to identify the key determinants that influence medical students’ acceptance of AI chatbots, thereby providing a basis for enhancing their integration into medical education. Understanding these factors is crucial for educators, policy makers, and technology developers to design and implement effective AI-driven educational tools that align with the needs and expectations of future health care professionals.

**Methods:**

A web-based electronic survey questionnaire was developed and distributed via social media to medical students across the country. The UTAUT was used as a theoretical framework to design the questionnaire and analyze the data. The relationship between behavioral intention to use AI chatbots and UTAUT predictors was examined using multivariable regression.

**Results:**

A total of 693 participants were from 57 universities covering 21 provinces or municipalities in China. Only a minority (199/693, 28.72%) reported using AI chatbots for studying, with ChatGPT (129/693, 18.61%) being the most commonly used. Most of the participants used AI chatbots for quickly obtaining medical information and knowledge (631/693**,** 91.05%) and increasing learning efficiency (594/693, 85.71%). Utilization behavior, social influence, facilitating conditions, perceived risk, and personal innovativeness showed significant positive associations with the behavioral intention to use AI chatbots (all *P* values were <.05).

**Conclusions:**

Chinese medical students hold positive perceptions toward and high intentions to use AI chatbots, but there are gaps between intention and actual adoption. This highlights the need for strategies to improve access, training, and support and provide peer usage examples to fully harness the potential benefits of chatbot technology.

## Introduction

The rapid advancements in artificial intelligence (AI) have significantly transformed various sectors, including health care. Among these advancements, AI chatbots have emerged as a promising tool with potential applications in medical education [1]. These intelligent systems use natural language processing and machine learning algorithms to engage in human-like dialogues, providing information in an understandable, efficient, interactive, and scenario-specific format, such as ChatGPT, Claude, Google Bard, and Bing’s AI [2]. The chatbots can assist medical students in medical research support, personalized learning, comprehending complex medical topics, developing clinical decision-making skills, and so forth [1,3]. A recent study demonstrated the efficacy of AI chatbots in answering complex medical questions and providing valuable medical educational support [4].

In China, integrating AI technology into medical education is particularly important, given the country’s substantial investment in AI development and its growing emphasis on innovative educational methodologies [5,6]. AI chatbots would facilitate personalized learning experiences when facing the situation of rigorous curricula and high student to teacher ratios in China. Medical students are a crucial target group for AI chatbot technology, as they are early adopters of technology and future health care providers who will play a vital role in shaping the future of health care. While research on AI chatbot applications in medical students has emerged [7-10], the utilization of, perceptions on, and intention to use AI chatbots among Chinese medical students are still unknown.

The adoption and effective utilization of AI chatbots among medical students depend on various factors, including their perceptions, attitudes, and behavioral intentions. The Unified Theory of Acceptance and Use of Technology (UTAUT), developed by Venkatesh et al [11], provides a comprehensive framework to understand the determinants of technology acceptance and usage, which is widely used in health care. Applying the UTAUT model in the context of AI chatbots can yield valuable insights into the factors that drive or hinder their adoption among medical students.

This study aims to explore the utilization of, perceptions on, and intention to use generative AI chatbots among medical students in China, using the UTAUT framework. By conducting a national cross-sectional survey, we seek to identify the key determinants that influence medical students’ acceptance of AI chatbots, thereby providing a basis for enhancing their integration into medical education. Understanding these factors is crucial for educators, policy makers, and technology developers to design and implement effective AI-driven educational tools that align with the needs and expectations of future health care professionals.

## Methods

### Participants and Procedure

The target population was medical students enrolled in Chinese medical colleges or universities. An electronic survey was developed through a web-based survey platform named Wenjuanxing Questionnaire Star (Ranxing Technology Corp.), and the survey link was distributed via WeChat (Tencent Holdings Ltd) to medical college students across the country. Using a convenience sampling method, the questionnaire was posted on WeChat Moments and sent to WeChat groups from the research team’s WeChat accounts. We identified relevant WeChat groups that consisted of medical students across various regions in China. These groups were selected based on their active participation in medical education discussions and their membership of medical students from diverse backgrounds and institutions. The research team directly contacted a total of 15 WeChat groups. To further enhance the reach, we used a snowball sampling method by requesting initial respondents to forward the survey link to other medical students in their network. Questionnaires that were considered valid included only the following: (1) each account responded only once, and (2) the total response time for completing the questionnaire was more than 300 seconds. Participants were recruited between June 2023 and July 2023.

To ensure adequate statistical power and precision for the intended analyses, we conducted a sample size calculation using G*Power software (version 3.1.9.7) [12]. The calculation was based on the following parameters: a small effect size (*f*_2_=0.05) was chosen for the multivariable regression analysis; the number of predictors was set at 15; the desired statistical power was set at 0.95; and the significance level was set at .05 (2-tailed). Based on these parameters, the minimum required sample size for the multivariable regression analysis was calculated to be 566 participants. However, to account for 20% missing data and increase the generalizability of the findings, we aimed to recruit 680 participants. Ultimately, we were able to collect 715 questionnaires across China, with 693 determined valid, representing a 96.9% final response rate.

### The Theoretical Framework

The study used the UTAUT as a theoretical framework for the research. The UTAUT describes 4 key independent variables: performance expectancy (PE), effort expectancy (EE), social influence (SI), and facilitating conditions (FC). In this study, PE measured the participants’ expectation that an AI chatbot will be useful for the study; EE measured the expectation that an AI chatbot is user-friendly and easy to use; SI measured the degree to which a user perceives that important others believe that he or she should use the new technology; and FC measured the degree to which a user believes that an organizational and technical infrastructure exists to support AI chatbot use [13]. The dependent variable BI was determined by PE, EE, SI, and FC. BI measured participants’ intention to use the AI chatbot in their future study.

The intention was used as an outcome instead of the actual use of an AI chatbot, because the application of AI chatbot services has not been widely commercialized in China. Most medical students may not have experience with an AI chatbot in their study. Also, BI is a good representation of actual behavior [14]. Previous studies confirmed that these 4 variables (PE, EE, SI, and FC) have a positive influence on the intention to use the AI technology [15-17]. The original UTAUT validation study found that the UTAUT model is robust in explaining a high degree of variance (70%) in BI [13]. Moderating effects of age, gender, and experience were not tested in this study.

In addition, we added perceived risk (PR), resistance bias (RB), and personal innovativeness (PI) as 3 variables to the original UTAUT model. PR is defined as the potential for loss in the pursuit of the desired outcome of using a technology and identified for 7 facets of PR [18]. Here, PR was measured for performance risk, time risk, and privacy risk. RB is resistance to change, referring to people’s attempts to maintain previous behaviors or habits that are connected to their past experiences when facing changes [16]. PI was designed to measure an individual’s willingness to try out any new information technology [19]. Since AI chatbots are an emerging technology in health care, a user’s inherent innovativeness may impact his or her intention to adopt this innovation, and some users may be accompanied by concerns and resistance to change when embracing the new technology. Previous studies found that PR and RB have been regarded as major barriers to health care information technology adoption [20,21], and PI has been statistically significant in predicting the BI of the user [22].

### Questionnaire and Instrument

The developed questionnaire consisted of 3 parts (see questionnaire in Multimedia Appendix 1): (1) participants’ sociodemographic information, such as age, gender, and grade level; (2) participants’ cognition of, attitude toward, and experience with AI chatbots (these items were designed as categorical variables and were derived through a comprehensive process, including literature review and expert consultation, to ensure their relevance and clarity); and (3) the scale of the research model. The model covered 7 constructs with 29 questionnaire items (Table 1). The items of the survey were ordered such that items measuring each construct were grouped. The responses were recorded using a 5-point Likert scale (ranging from 1=totally disagree to 5=totally agree) in which the higher score values indicated a higher level of a construct and a higher score of the outcome (BI) indicated greater intention to use the AI chatbot.

In the third part of the questionnaire, each item in the scale was sourced from relevant literature related to new technology acceptance research. The main modifications to the original instrument were made to fit the context of an AI chatbot used for medical students, such as changing the word “system” to “AI Chatbot.” The items that assessed PE, EE, SI, FC, and BI were adopted from the original instrument developed by Venkatesh et al [11]. The original survey was validated and applied to previous studies based on the UTAUT model [16,23,24]. The items that assessed PR and RB were adopted from the validated questionnaire developed by Zhai et al [16]. The reliability of the items’ scales was tested by Cronbach α coefficient analysis. The results of Cronbach α are considered to have acceptable reliability (Table 1), as the generally accepted rule is that α values of 0.6‐0.7 indicate an acceptable level of reliability, and 0.8 or greater is a very good level [25].

After we developed the questionnaire and before implementing the survey, we conducted a consensus panel of 5 experts to review the questionnaire and ensure clarity of the survey and content validity. We then conducted a pilot study of 20 students to clarify phrasing and eliminate items that were not identifiable in the questionnaire.

**Table 1.** The model constructs and its measuring scale items.

Constructs and items		Cronbach α
**PE** ^**a**^		0.920
	PE 1: I would find AI^b^ Chatbot useful in my study.	
	PE 2: Using AI Chatbot will enable me to accomplish tasks more quickly.	
	PE 3: Using AI Chatbot will increase my productivity.	
	PE 4: If I use AI Chatbot, I will increase my chances of getting better grades.	
**EE** ^**c**^		0.904
	EE 1: My interaction with AI Chatbot will be clear and understandable.	
	EE 2: It would be easy for me to become skillful at using AI Chatbot.	
	EE 3: I would find AI Chatbot easy to use.	
	EE 4: Learning to operate AI Chatbot is easy for me.	
**SI** ^**d**^		0.871
	SI 1: People who influence my behavior (eg, classmates, colleagues, and friends) think that I should use AI Chatbot.	
	SI 2: People who are important to me (eg, department heads, supervisors, and hospital leaders) think that I should use AI Chatbot.	
	SI 3: The senior health administration has been helpful in the use of AI Chatbot.	
	SI 4: In general, my university and hospital have supported the use of AI Chatbot.	
**FC** ^**e**^		0.756
	FC 1: I have the resources necessary to use AI Chatbot.	
	FC 2: I have the knowledge necessary to use AI Chatbot.	
	FC 3: AI Chatbot is not compatible with other systems I use.	
	FC 4: A specific person (or group) is available for assistance with the AI Chatbot difficulties.	
**PR** ^**f**^		0.643
	PR 1: There is a possibility of malfunction and performance failure, so the AI Chatbot fails to deliver accurate information and could mislead my study.	
	PR 2: There is a probability that I need more time to fix the errors and nuances of the AI Chatbot.	
	PR 3: I am worried that AI chatbots will reveal my private information.	
**RB** ^**g**^		0.879
	RB 1: I do not want AI chatbots to change the way I study or work because the new AI tools are unfamiliar to me.	
	RB 2: I do not want to use the AI chatbots because of past experiences; these new high-tech products always fall flat during practical application.	
	RB 3: I do not want to use the AI chatbots because there is a possibility of losing my job, as artificial intelligence–assisted technology may do my work better than me.	
**PI** ^**h**^		0.634
	PI 1: If I heard about a new information technology, I would look for ways to experiment with it.	
	PI 2: Among my peers, I am usually the first to try out new information technologies.	
	PI 3: In general, I am hesitant to try out new information technologies.	
	PI 4: I like to experiment with new information technologies.	
**BI** ^**i**^		0.946
	BI 1: I intend to use the AI chatbots in the next 2 months.	
	BI 2: I predict I would use the AI chatbots in the next 2 months.	
	BI 3: I plan to use the AI chatbots in the next 2 months.	

a PE: performance expectancy.

b AI: artificial intelligence.

c EE: effort expectancy.

d SI: social influence.

e FC: facilitating conditions.

f PR: perceived risk.

g RB: resistance bias.

h PI: personal innovativeness.

i BI: behavioral intention.

### Data Analysis

The statistical software SPSS 25.0 (IBM Corp) was used to calculate the Cronbach α coefficient. Data analysis was carried out using descriptive statistics, such as means, frequencies, and percentages, as well as inferential statistics, such as multiple linear regression, to explore the relationships between the dependent variable (BI) and the set of predictors (PE, EE, SI, FC, PR, RB, and PI). The α level was set at .05 for all analyses. Data analysis was performed using Stata (version 17.0; StataCorp LLC).

### Ethical Considerations

Ethical approval was obtained from the Ethics Committee on Biomedical Research, West China Hospital of Sichuan University (approval number: 2023‐834). The research purpose; methods; and participants’ rights, including that they could cease participation at any point without penalty, were explained. All the participants read and signed the electronic informed consent before completing the questionnaire. The detailed information on the informed consent form is given in the questionnaire in Multimedia Appendix 1. This survey was anonymous and voluntary. To promote survey completion and ensure an adequate response rate, postsurvey gifts were randomly raffled as an incentive.

## Results

### Participants’ Information

**Figure 1.**
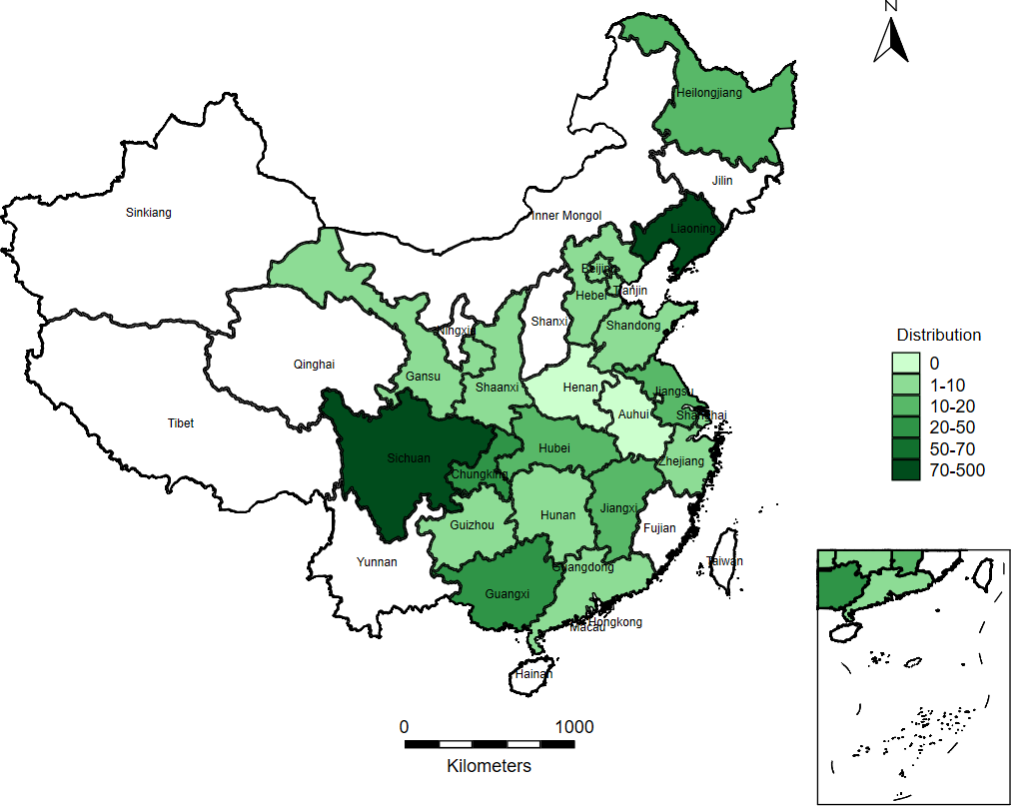
Sample distribution.

A total of 693 participants were from 57 universities covering 21 provinces or municipalities. The sample distribution is shown in Figure 1. The demographic characteristics of the participants are shown in Table 2. The majority of participants (251/693, 63.78%) were female, while 36.22% (442/693) were male. The average age was 22.6 (SD 5.2) years, and more than half of the participants were in the 20‐ to 24-year age range (413/693, 59.60%). The majority (543/693, 78.35%) were undergraduate students. The mean self-reported academic score was 73.7 (SD 14.8), and the most common self-reported score range was 80‐89 (247/693, 35.64%).

**Table 2.** Demographic characteristics of participants (N=693).

Characteristics	Participants, n (%)	
**Sex**		
Male	251 (36.22)	
Female	442 (63.78)	
**Age (years)**		
＜20	141 (20.35)	
20‐24	413 (59.60)	
25‐29	87 (12.55)	
≥30	52 (7.50)	
**Hukou type** ^**a**^		
Urban	364 (52.53)	
Rural	329 (47.47)	
**Education level**		
Undergraduate	543 (78.35)	
Master student	101 (14.57)	
Doctor student	49 (7.07)	
**Self-reported academic scores^b^**		
90‐100	70 (10.10)	
80‐89	247 (35.64)	
70‐79	163 (23.52)	
60‐69	144 (20.78)	
＜60	69 (9.96)	

a “Hukou type” refers to the classification within the Chinese household registration system. This system classifies individuals based on their place of household registration and typically includes 2 main categories: Urban Hukou and Rural Hukou.

b “Academic scores” for medical students refer to the grades or marks they receive in various courses throughout their medical education in college or university. Academic scores are important indicators of a student’s academic performance, reflecting his or her learning effectiveness.

### Participants’ Perception of AI Chatbots

Table 3 presents the participants’ cognition, attitudes, usage behavior, and willingness to pay for AI chatbots. While only 24.68% (171/693) of participants reported being fairly familiar with AI chatbots and 4.47% (31/693) were very familiar with AI chatbots, 59.88% (415/693) agreed or strongly agreed with using them for study or work purposes. Of the 28.72% (199/693) who have used AI chatbots for studying, mainly ChatGPT (129/693, 18.61%), 50.25% (100/199) reported occasional usage as needed. Among nonusers, 55.06% (272/494) expressed willingness to learn AI chatbot usage, with the main reasons for unwillingness being no need (15/29, 51.72%) and no interest (15/29, 51.72%). In addition, 36.45% (242/664) preferred to use AI chatbots without charge.

Table 4 summarizes the participants’ purposes for using AI chatbots and their perceived advantages and disadvantages. The primary purposes were quickly obtaining medical information and knowledge (631/693, 91.05%) and increasing learning efficiency (594/693, 85.71%). Perceived advantages included effectively helping medical students learn (631/693, 91.05%) and providing fast and accurate medical information (624/693, 90.04%). However, data privacy breaches (635/693, 91.63%) and risks of misdiagnosis or underdiagnosis (619/693, 89.32%) were predominant concerns.

**Table 3.** Participants’ cognition, attitude, usage behavior, and willingness to pay for AI chatbots (exchange rate: US $1=¥7.22, July 9, 2023).

Items	Participants, n (%)	
**Do you know what an AI^a^ chatbot is? (N=693)**		
Completely unfamiliar	17 (2.45)	
Unfamiliar	103 (14.86)	
Average	371 (53.54)	
Fairly familiar	171 (24.68)	
Very familiar	31 (4.47)	
**Do you agree with the use of AI chatbot applications for study or work? (N=693)**		
Strongly disagree	16 (2.31)	
Disagree	31 (4.47)	
Neutral	231 (33.33)	
Agree	328 (47.33)	
Strongly agree	87 (12.55)	
**Have you used AI chatbots in your study? (N=693)**		
No	494 (71.28)	
**Yes**	199 (28.72)	
ChatGPT	129 (18.61)	
New Bing	20 (2.89)	
Others	31 (4.47)	
Missing	19 (2.74)	
**How often do you use this AI chatbot? (N=199)**		
Every day	18 (9.05)	
Several times a week	36 (18.09)	
About once a week	6 (3.02)	
Occasionally, as needed	100 (50.25)	
Rarely, only in specific situations	39 (19.60)	
**If you have not used it, would you be willing to learn how to use AI chatbots? (N=494)**		
Strongly unwilling	6 (1.21)	
Unwilling	23 (4.66)	
Neutral	193 (39.07)	
Somewhat willing	217 (43.93)	
Very willing	55 (11.13)	
**If you are unwilling to use AI chatbots, what is the main reason? (N=29)**		
No need	15 (51.72)	
No interest	15 (51.72)	
Inconvenient operation	8 (27.59)	
Worries about privacy issues	14 (48.28)	
Worries about inaccurate information provided	13 (44.83)	
**If a high-quality and convenient AI chatbot were available to assist you in your learning, how much would you be willing to pay per month to use it? (N=664)**		
Free	242 (36.45)	
<¥20	188 (28.31)	
¥20 to ¥50	150 (22.59)	
¥50 to ¥100	59 (8.89)	
>¥100	25 (3.77)	

a AI: artificial intelligence.

**Table 4.** Participants’ purpose of using artificial intelligence (AI) chatbots and perceived advantages or disadvantages.^a^

Items		Total choices, n (%)	First choice, n (%)
**What is your main purpose in using an AI chatbot?**			
	Quickly obtaining basic medical information and knowledge	631 (91.05)	434 (62.63)
	Increasing learning efficiency.	594 (85.71)	69 (9.96)
	Seeking answers and guidance for complex medical questions.	583 (84.13)	68 (9.81)
	Exploring new research and academic resources.	564 (81.39)	43 (6.20)
	Self-health management and self-diagnosis.	520 (75.04)	22 (3.17)
	Improving the experience of medical learning and training.	509 (73.45)	12 (1.73)
	Retrieving various information, such as regular search engines.	443 (63.92)	30 (4.33)
	Chatting and entertainment.	377 (54.40)	12 (1.73)
	Others^b^	—^c^	—
**What advantages do you think AI chatbots have?**			
	They can effectively help medical students learn and master medical knowledge.	631 (91.05)	157 (22.66)
	They can provide fast and accurate medical information and diagnosis results.	624 (90.04)	405 (58.44)
	They can improve the efficiency and quality of health care services.	575 (82.97)	53 (7.65)
	They can reduce the workload and burden of doctors.	574 (82.83)	72 (10.39)
	Others^d^	—	—
**What disadvantages or risks do you think AI chatbots have?**			
	There may be risks of data privacy breaches.	635 (91.63)	406 (58.59)
	There may be risks of misdiagnosis or underdiagnosis.	619 (89.32)	137 (19.77)
	They may potentially lead to the degradation or unemployment of medical professionals.	570 (82.25)	87 (12.55)
	They may potentially reduce the personal touch and humanization of health care services.	559 (80.66)	58 (8.37)
	Others^e^	—	—

a The questions in this part of the survey were ranking questions. “Total choices” provide an overall measure of how often an option was selected. “First choice” represents the preference for an option as the most preferred or prioritized choice among respondents.

b Response examples: “Make code modifications,” “Polishing the content of the documents,” “Complete some unimportant homework,” and “Online operation training.”

c Not applicable.

d Response examples: “Reduce feelings of loneliness,” “Regulate emotions of healthcare workers,” “Provide arguments for the group work,” and “Provide timely and patient answers.”

e Response examples: “Provide misleading information, such as fabricating references,” “Patients may have doubts and mistrust towards these technological products,” “AI currently cannot reflect the artistic elements required in medicine,” and “It may not be able to provide the desired, high-quality answers.”

### Descriptive Statistics of the UTAUT Constructs

**Figure 2.**
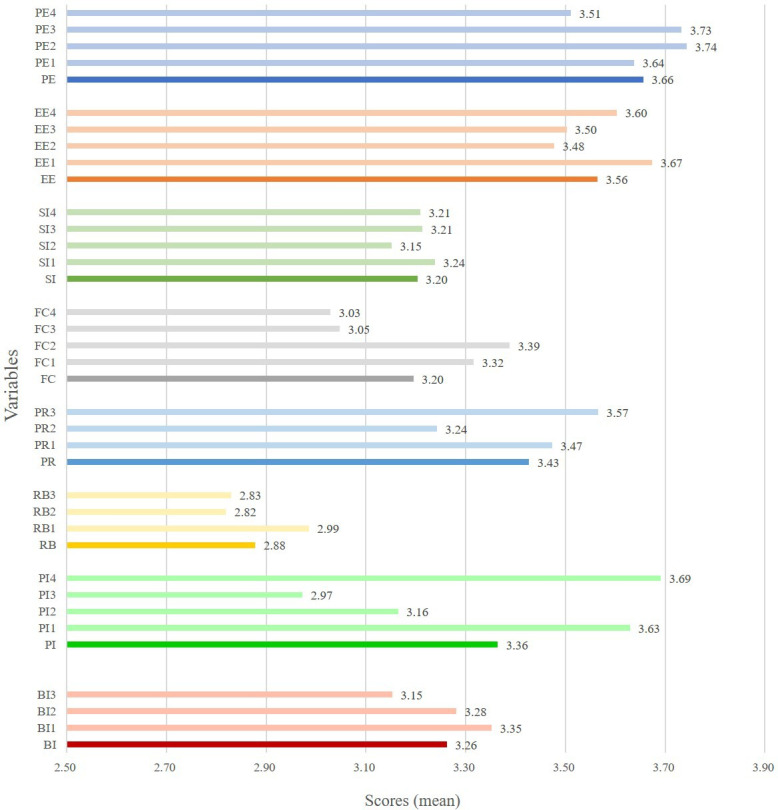
Descriptive statistics of the Unified Theory of Acceptance and Use of Technology constructs. BI: behavioral intention; EE: effort expectancy; FC: facilitating conditions; PE: performance expectancy; PI: personal innovativeness; PR: perceived risk; RB: resistance bias; SI: social influence.

Descriptive statistics (mean [SD]) were reported to explain and describe the UTAUT constructs (Table S1 in Multimedia Appendix 1). The value of each construct ranges from 1 to 5 (1=strongly disagree, 5=strongly agree). As shown in Figure 2, the mean for PE, EE, SI, FC, PR, and PI were higher than 3 and the mean for RB was <3. The highest score was PE at 3.66, followed by EE at 3.56. The mean of BI was 3.26, which shows a higher level of intention to use AI chatbots among Chinese medical students.

### Determinant Factors of Intention to Use AI Chatbots

A multiple linear regression analysis was conducted to identify factors influencing medical students’ intentions to use AI chatbots (Table 5). Utilization behavior (β=.27; *P*<.001), SI (β=.32; *P*<.001), FC (β=.29; *P*<.001), PR (β=.27; *P*<.001), and PI (β=.35; *P*<.001) were significantly positively associated with BI. PI had the largest positive regression coefficient (β=.35) compared with the other significant variables. PE (β=.09; *P*=.12), EE (β=.03; *P*=.60), and RB (β=−0.04; *P*=.32) did not significantly affect BI.

**Table 5.** Analysis of influence factors of medical students’ behavioral intention to use artificial intelligence chatbots.^a^

Variables	Coefficient	SE	*t*	*P* value	95% CI
Age (years)	0.01	0.01	1.89	.06	0.00 to 0.02
Gender	−0.04	0.05	−0.83	.41	−0.15 to 0.06
Hukou type	0.10	0.05	1.93	.06	0.00 to 0.20
Education level	−0.02	0.05	−0.46	.65	−0.12 to 0.08
Academic scores	0.00	0.00	0.51	.61	0.00 to 0.00
Cognition^b^	0.01	0.04	0.27	.79	−0.06 to 0.08
Attitude^c^	0.03	0.04	0.89	.37	−0.04 to 0.11
Utilization behavior^d^	0.27	0.07	4.19	*<*.001	0.15 to 0.40
Performance expectancy	0.09	0.06	1.56	.12	−0.02 to 0.21
Effort expectancy	0.03	0.06	0.53	.60	−0.08 to 0.14
Social influence	0.32	0.05	6.32	<.001	0.22 to 0.42
Facilitating conditions	0.29	0.07	4.13	*<.*001	0.15 to 0.42
Perceived risk	0.27	0.05	5.33	*<.*001	0.17 to 0.37
Resistance bias	−0.04	0.04	−0.99	.32	−0.12 to 0.04
Personal innovativeness	0.35	0.07	5.33	*<.*001	0.22 to 0.48
Constant term	−1.98	0.31	−6.31	*<.*001	−2.60 to −1.36

a Model parameters: Probability>*F*=0, *R*2=0.518, adjusted *R*2=0.507, and Root Mean Square Error=0.651. df_Total=692, df_Model=15, df_Residual=677. The results of multicollinearity diagnostics showed that there is no multicollinearity among all independent variables in the multiple linear regression (Table S2 in Multimedia Appendix 1).

b The variable “Cognition” is measured by “Do you know what an AI chatbot is?”

c The variable “Attitude” is measured by “Do you agree with the use of AI chatbot applications for study or work?”

d The variable “Utilization behavior” is measured by “Have you used AI chatbots in your study?”

## Discussion

### Principal Findings

In this study, we examined the perceptions of Chinese medical students toward Natural Language Processing–based AI chatbots and investigated the factors that may influence their intention to use such technology based on the UTAUT model. This research yielded several key findings. First, the medical students demonstrated positive perceptions and expressed a high BI to use AI chatbots. Second, among the factors considered, SI and FC emerged as more influential in the adoption of AI chatbots among medical students than PE and EE. However, PE and EE were not found to have a significant relationship with BI. Third, PR and PI positively influenced BI, while RB did not show a significant association with BI.

This study revealed that although most medical students have limited knowledge about AI chatbots at an early time, they hold positive perceptions and demonstrate a strong intention to use this innovative technology. The overall sample displayed high BIs, with a mean score of 3.26 out of 5.00. Furthermore, 81.63% (1697/2079) of participants rated their intention as 3 or higher, indicating their plans to use the technology within the next 2 months. These findings align with previous research indicating that while medical students may lack knowledge about AI and its applications, they maintain a favorable view of AI in the medical field and are willing to adopt it [26,27]. The majority of participants believe that AI chatbots have the potential to enhance their study or work performance, improve efficiency, and provide fast and accurate medical information, among other benefits. However, limited availability and coverage of AI chatbots in China have resulted in less than one-third of participants actually using these tools and only a few using them on a daily basis. This indicates a gap between intention to use and actual adoption. Practical barriers, such as inadequate technical infrastructure and lack of support, may hinder the actual implementation and use of AI chatbots. In addition, our regression analysis revealed that utilization behavior significantly influences medical students’ intentions to use AI chatbots. User experience may impact their perceptions of the technology from multiple aspects, thereby affecting their usage intentions.

This study found that SI and FC have a stronger impact on BI than PE and EE. This finding aligns with research examining the perceptions of Chinese radiation oncologists toward adopting AI-assisted contouring technology [16]. However, it contradicts some prior studies that have established a positive and significant relationship between PE and EE with students’ BI to use AI-assisted learning environments [28] or chatbots [29]. This suggests that factors such as PE and EE may be less critical for the population in this study, although they rated PE and EE higher than other dimensions. It is possible that as medical students are still in training, they rely more on the experiences of their peers and the infrastructure provided by their educational institution to guide their technology use. Thus, demonstrating adoption and endorsement from fellow students, professors, and the academic medical system may be more influential in persuading them to use AI chatbots than emphasizing use and usability. Ensuring accessibility within the educational context appears to shape students’ willingness to use AI chatbots more than their individual perceptions of performance and efficiency.

Interestingly, this study found that PR and PI positively influenced the intentions of medical students to use AI chatbots, while RB was not found to be a significant factor. This suggests that concerns regarding the risks associated with adopting AI chatbots were outweighed by the students’ openness to embracing new technologies. Those with a greater inclination toward innovation recognized the potential benefits despite the potential risks involved. This finding aligns with previous research indicating that perceived usefulness can override PR when it comes to determining acceptance of technology [18]. It also reflects a growing understanding that AI systems present both opportunities and risks, necessitating ethical analysis and oversight [30], including privacy breaches and the possibility of misinformation. Notably, we found that PI emerged as a key determinant of user behavior intentions, which is consistent with a similar study [31]. However, RB did not negatively impact intentions, suggesting that medical students may have fewer biases against AI than health care professionals in hospitals who may fear a loss of professional autonomy and challenges in integrating AI into clinical workflows [32]. Encouraging PI while addressing risk concerns through testing and regulation may further bolster the adoption of AI chatbots.

### Implications for Practice

Based on our findings, we recommend the following specific strategies for educational institutions and AI chatbot developers to enhance the adoption rate among medical students. First, medical schools and health care organizations should prioritize efforts to improve FC and leverage SI to drive the adoption of AI chatbots, rather than solely focusing on performance benefits. Providing integrated access, training, and IT support and sharing peer usage examples can help translate positive intentions into actual usage behaviors. In addition, demonstrating value through pilot studies and addressing valid risk concerns will promote responsible and open adoption of the technology. Targeted training in AI competencies can further equip students to become champions of safe and effective adoption. The key lies in creating optimal environments and processes to enable the proficient use of AI systems such as chatbots as students transition into practice.

### Strengths and Limitations

This study was the first to use the UTAUT theoretical framework to analyze medical students’ intention to use AI chatbots. It possesses several strengths, including the robust technology adoption model used, the focus on an important user population, and the identification of key variables influencing intentions. However, there are some limitations that need to be addressed in future studies. First, the unbalanced research sample primarily from Sichuan province may limit the generalizability of the findings, potentially overrepresenting specific regional experiences. Although we distributed the survey widely, future studies should use stratified sampling for better regional representation. Second, the cross-sectional design offers only a snapshot of adoption, which may change over time as participants accumulate knowledge and experience. Future research should consider longitudinal designs to track these changes. Third, it is crucial to acknowledge that the field of AI chatbots is rapidly evolving, and our findings capture perceptions and attitudes at a specific point in time. As AI chatbot capabilities continue to advance, the external validity of our findings may need to be reevaluated.

Future studies with larger samples using longitudinal methods would enhance our understanding of actual perceptions and usage patterns over time. For example, a longitudinal study could follow a cohort of medical students from their entry into medical school until graduation, periodically assessing their perceptions, intentions, and actual usage of AI chatbots. This longitudinal approach would capture how their adoption and experiences with AI chatbots evolve as they progress through their medical education and gain more exposure to clinical settings. Furthermore, mixed methods designs, combining quantitative surveys with qualitative interviews or focus groups, could provide more in-depth insights into specific barriers, challenges, and facilitators influencing AI chatbot adoption among medical students. Overall, this study lays the foundation for a wide range of future research, which can deepen knowledge and generate evidence to guide the implementation of AI in education and health care.

### Conclusions

This study offers valuable insights into medical students’ utilization of, perceptions on, and intention to use AI chatbots in health care. The results indicate that these medical students have positive perceptions and strong intentions to use chatbots, primarily influenced by SI and FC rather than PE and EE. However, despite these intentions, there remains a gap between intention and actual adoption, signaling the need for strategies that improve access, training, and support and provide peer usage examples to enhance the realization of the potential benefits of chatbots. While concerns about risks exist, the students’ general openness to innovation suggests that the integration of AI with proper oversight is well received. As future health care professionals, students serve as early adopters who can shape wider acceptance if barriers to adoption are actively addressed. This research provides a foundation for understanding the technology needs and motivations of this important user population in order to guide the successful implementation of AI.

## Supplementary material

10.2196/57132 Multimedia Appendix 1 Survey questionnaire on medical students’ use of artificial intelligence chatbots (translated version), descriptive statistics of the Unified Theory of Acceptance and Use of Technology constructs, and multicollinearity diagnostics.

## Abbreviations

AI: artificial intelligence
BI: behavioral intention
EE: effort expectancy
FC: facilitating conditions
PE: performance expectancy
PI: personal innovativeness
PR: perceived risk
RB: resistance bias
SI: social influence
UTAUT: Unified Theory of Acceptance and Use of Technology

## References

[1] Ghorashi N, Ismail A, Ghosh P, Sidawy A, Javan R (2023). AI-powered chatbots in medical education: potential applications and implications. Cureus.

[2] Xie Y, Seth I, Hunter-Smith DJ, Rozen WM, Seifman MA (2024). Investigating the impact of innovative AI chatbot on post-pandemic medical education and clinical assistance: a comprehensive analysis. ANZ J Surg.

[3] Wu Y, Zheng Y, Feng B, Yang Y, Kang K, Zhao A (2024). Embracing ChatGPT for medical education: exploring its impact on doctors and medical students. JMIR Med Educ.

[4] Baglivo F, De Angelis L, Casigliani V, Arzilli G, Privitera GP, Rizzo C (2023). Exploring the possible use of AI chatbots in public health education: feasibility study. JMIR Med Educ.

[5] (2017). The State Council’s notice on issuing the next generation artificial intelligence development plan. The State Council of the People’s Republic of China.

[6] (2024). The Ministry of Education releases four initiatives to promote artificial intelligence empowerment in education. Xinhua News Agency.

[7] Han JW, Park J, Lee H (2022). Analysis of the effect of an artificial intelligence chatbot educational program on non-face-to-face classes: a quasi-experimental study. BMC Med Educ.

[8] Suárez A, Adanero A, Díaz-Flores García V, Freire Y, Algar J (2022). Using a virtual patient via an artificial intelligence chatbot to develop dental students’ diagnostic skills. Int J Environ Res Public Health.

[9] Moldt JA, Festl-Wietek T, Madany Mamlouk A, Nieselt K, Fuhl W, Herrmann-Werner A (2023). Chatbots for future docs: exploring medical students’ attitudes and knowledge towards artificial intelligence and medical chatbots. Med Educ Online.

[10] Tangadulrat P, Sono S, Tangtrakulwanich B (2023). Using ChatGPT for clinical practice and medical education: cross-sectional survey of medical students’ and physicians’ perceptions. JMIR Med Educ.

[11] Venkatesh V, Morris MG, Davis GB, Davis FD (2003). User acceptance of information technology: toward a unified view. MIS Q.

[12] Faul F, Erdfelder E, Buchner A, Lang AG (2009). Statistical power analyses using G*Power 3.1: tests for correlation and regression analyses. Behav Res Methods.

[13] Ammenwerth E (2019). Technology acceptance models in health informatics: TAM and UTAUT. Stud Health Technol Inform.

[14] Eccles MP, Hrisos S, Francis J (2006). Do self- reported intentions predict clinicians’ behaviour: a systematic review. Implement Sci.

[15] Jain R, Garg N, Khera SN (2022). Adoption of AI-enabled tools in social development organizations in India: an extension of UTAUT model. Front Psychol.

[16] Zhai H, Yang X, Xue J (2021). Radiation oncologists’ perceptions of adopting an artificial intelligence–assisted contouring technology: model development and questionnaire study. J Med Internet Res.

[17] García de Blanes Sebastián M, Sarmiento Guede JR, Antonovica A (2022). Application and extension of the UTAUT2 model for determining behavioral intention factors in use of the artificial intelligence virtual assistants. Front Psychol.

[18] Featherman MS, Pavlou PA (2003). Predicting e-services adoption: a perceived risk facets perspective. Int J Hum Comput Stud.

[19] Agarwal R, Prasad J (1998). A conceptual and operational definition of personal innovativeness in the domain of information technology. Inf Syst Res.

[20] Bhattacherjee A, Hikmet N (2007). Physicians’ resistance toward healthcare information technology: a theoretical model and empirical test. Eur J Inf Syst.

[21] Hsieh PJ (2015). Physicians’ acceptance of electronic medical records exchange: an extension of the decomposed TPB model with institutional trust and perceived risk. Int J Med Inform.

[22] Simarmata MTA, Hia IJ (2020). The role of personal innovativeness on behavioral intention of information technology. J Econ Bus.

[23] Kijsanayotin B, Pannarunothai S, Speedie SM (2009). Factors influencing health information technology adoption in Thailand’s community health centers: applying the UTAUT model. Int J Med Inform.

[24] Alabdullah JH, Van Lunen BL, Claiborne DM, Daniel SJ, Yen CJ, Gustin TS (2020). Application of the unified theory of acceptance and use of technology model to predict dental students’ behavioral intention to use teledentistry. J Dent Educ.

[25] Ursachi G, Horodnic IA, Zait A (2015). How reliable are measurement scales? External factors with indirect influence on reliability estimators. Proc Econ Finance.

[26] Ahmed Z, Bhinder KK, Tariq A (2022). Knowledge, attitude, and practice of artificial intelligence among doctors and medical students in Pakistan: a cross-sectional online survey. Ann Med Surg.

[27] Mousavi Baigi SF, Sarbaz M, Ghaddaripouri K, Ghaddaripouri M, Mousavi AS, Kimiafar K (2023). Attitudes, knowledge, and skills towards artificial intelligence among healthcare students: a systematic review. Health Sci Rep.

[28] Wu W, Zhang B, Li S, Liu H (2022). Exploring factors of the willingness to accept AI-assisted learning environments: an empirical investigation based on the UTAUT model and perceived risk theory. Front Psychol.

[29] Almahri FAJ, Bell D, Merhi M Understanding student acceptance and use of chatbots in the United Kingdom universities: a structural equation modelling approach.

[30] Morley J, Floridi L, Kinsey L, Elhalal A (2020). From what to how: an initial review of publicly available AI ethics tools, methods and research to translate principles into practices. Sci Eng Ethics.

[31] Tian W, Ge J, Zhao Y, Zheng X (2024). AI chatbots in Chinese higher education: adoption, perception, and influence among graduate students-an integrated analysis utilizing UTAUT and ECM models. Front Psychol.

[32] Lambert SI, Madi M, Sopka S (2023). An integrative review on the acceptance of artificial intelligence among healthcare professionals in hospitals. NPJ Digit Med.

