# Supplementary material for: Utilization of, Perceptions on, and Intention to Use AI Chatbots Among Medical Students in China: National Cross-Sectional Study
Source: JMIR Med Educ. 2024 Oct 28;10:e57132. doi: 10.2196/57132 (PMC11533383; doi:10.2196/57132)
Supplement: Multimedia Appendix 1 [file mededu-v10-e57132-s001.docx]

**Supplementary file**

Table S1 Descriptive statistics of the UTAUT constructs

| Variable | Mean | Std. dev. | Min | Max |
| --- | --- | --- | --- | --- |
| PE1 | 3.64 | 0.78 | 1 | 5 |
| PE2 | 3.74 | 0.75 | 1 | 5 |
| PE3 | 3.73 | 0.78 | 1 | 5 |
| PE4 | 3.51 | 0.80 | 1 | 5 |
| PE | 3.66 | 0.70 |  |  |
| EE1 | 3.67 | 0.76 | 1 | 5 |
| EE2 | 3.48 | 0.83 | 1 | 5 |
| EE3 | 3.50 | 0.81 | 1 | 5 |
| EE4 | 3.60 | 0.79 | 1 | 5 |
| EE | 3.56 | 0.70 |  |  |
| SI1 | 3.24 | 0.87 | 1 | 5 |
| SI2 | 3.15 | 0.88 | 1 | 5 |
| SI3 | 3.21 | 0.88 | 1 | 5 |
| SI4 | 3.21 | 0.82 | 1 | 5 |
| SI | 3.20 | 0.73 |  |  |
| FC1 | 3.32 | 0.90 | 1 | 5 |
| FC2 | 3.39 | 0.83 | 1 | 5 |
| FC3 | 3.05 | 0.95 | 1 | 5 |
| FC4 | 3.03 | 0.98 | 1 | 5 |
| FC | 3.20 | 0.48 |  |  |
| BI1 | 3.35 | 0.95 | 1 | 5 |
| BI2 | 3.28 | 0.97 | 1 | 5 |
| BI3 | 3.15 | 1.01 | 1 | 5 |
| BI | 3.26 | 0.93 |  |  |
| PR1 | 3.47 | 0.78 | 1 | 5 |
| PR2 | 3.24 | 0.89 | 1 | 5 |
| PR3 | 3.57 | 0.82 | 1 | 5 |
| PR | 3.43 | 0.64 |  |  |
| RB1 | 2.99 | 0.93 | 1 | 5 |
| RB2 | 2.82 | 0.96 | 1 | 5 |
| RB3 | 2.83 | 0.97 | 1 | 5 |
| RB | 2.88 | 0.86 |  |  |
| PI1 | 3.63 | 0.77 | 1 | 5 |
| PI2 | 3.16 | 0.84 | 1 | 5 |
| PI3 | 2.97 | 0.90 | 1 | 5 |
| PI4 | 3.69 | 0.76 | 1 | 5 |
| PI | 3.36 | 0.51 |  |  |

*Note*. PE = Performance expectancy,EE = Effort expectancy, SI = Social influence, FC = Facilitating conditions, PR = Perceived risk, RB = Resistance bias, PI = Personal innovativeness, BI = Behavioral intention

Table S2 Multicollinearity diagnostics

| Variables | VIF | Tolerance |
| --- | --- | --- |
| Age | 1.42 | 0.7021 |
| Gender | 1.08 | 0.9261 |
| Hukou type | 1.09 | 0.9148 |
| Education level | 1.46 | 0.6829 |
| Academic scores | 1.09 | 0.9167 |
| Cognition | 1.33 | 0.7515 |
| Attitude | 1.59 | 0.6288 |
| Utilization behavior | 1.43 | 0.7006 |
| Performance expectancy | 2.82 | 0.3546 |
| Effort expectancy | 2.65 | 0.3768 |
| Social influence | 2.23 | 0.4480 |
| Facilitating conditions | 1.81 | 0.5540 |
| Perceived risk | 1.74 | 0.5746 |
| Resistance bias | 1.98 | 0.5039 |
| Personal innovativeness | 1.90 | 0.5270 |

*Note*. VIF = Variance Inflation Factor. The value of the tolerance of each independent variable $>$ 0.20, the value of VIF of each independent $<$ 5. The results indicate that there is no multicollinearity among all independent variables in the multiple linear regression.

**Supplementary file**

Survey Questionnaire on Medical Students' Use of AI Chatbots

Dear peers,

Greetings! Firstly, thank you very much for taking 3-5 minutes to participate in this survey. The aim of this questionnaire is to understand the cognition, attitude, and willingness to use AI chatbots among medical students. This survey is conducted anonymously, there are no uniform answers to all questions, please answer truthfully according to your actual situation.

This survey has been approved by the Ethics Committee of ..University. The information you provide is of great significance to the development research of AI education for medical students in our country. All data will be strictly confidential and used only for scientific research. Your completion of this survey signifies that you have given informed consent to participate; if you have any questions, you can also choose to withdraw from this research, and this will not affect you in any way. After completing the analysis, we will publish the statistical results of the survey in a certain form, where you can understand the current situation of medical students using AI tools, so that you can better understand the academic environment you are in. If you wish to know more about the progress of the study, you can leave your email address at the end of the questionnaire.

For the purposes of this research, the survey targets students of medical-related majors in medical colleges or medical universities and medical schools of various comprehensive universities. If you are not a medical-related major, please ignore this invitation, for which we sincerely apologize.

If you have any questions related to this research, you can contact the researchers of this project.

We sincerely thank you again for your participation!

To express our gratitude, we will randomly select 15 students from all submitted questionnaires to receive small creative gifts from... If you wish to participate, please leave your email at the end of the questionnaire. We will start the random draw after the data collection is completed, and contact you via email regarding mailing matters.

Informed Consent Form

I have been informed about the background, purpose, steps, risks and benefits of the research on the use of AI chatbots, and have been given enough time to read the informed consent form, discuss it with others, and have my questions about the research answered; I have been informed that I can contact the researchers at any time with questions related to the research, and I can contact the project team members at any time with issues related to my rights/interests, and have obtained accurate contact information; I have been informed that I can withdraw from this research at any time without giving any reason. [Single choice question] *

○ Agree

○ Disagree (please jump to the end of the questionnaire and submit)

Prompt Information

AI chatbots are automated dialogue systems based on AI technology, capable of interacting and conversing with human users in real time. These robots utilize technologies such as natural language processing, machine learning, and deep learning to understand and generate natural language and adapt to user needs and preferences in conversations. (In this survey, AI chatbots refer to text-generating chatbots like ChatGPT, New Bing)

Part One

1. Your age: _______ years old [Fill in the blank] *

2. Your gender: [Single choice question] *

○Male ○Female

3. Your household registration type: [Single choice question] *

○Urban ○Rural

4. The district where your school is located: [Fill in the blank] *

_________________________________

5. Name of the school you are currently attending: ________ [Fill in the blank] *

6. What stage of education are you currently at? [Single choice question] *

○Undergraduate

○Masters

○Doctorate

7. What year are you in at your current level of education? [Single choice question] *

○First Year

○Second Year

○Third Year

○Fourth Year

○Fifth Year or Above

8. Your major: [Single choice question] *

○Basic Medical Science

○Clinical Medicine

○Oral Medicine

○Public Health and Preventive Medicine

○Traditional Chinese Medicine

○Special Medicine

○Medical Technology

○Nursing

○History of Science and Technology

○Veterinary Medicine Engineering

9. Please score your medical learning achievement based on your academic performance. Please choose a score within the range of 0 to 100 that best fits your self-evaluation, where 0 points means very poor, and 100 points means excellent.

______ points [Matrix text question] [Input a number between 0 and 100] *

Learning Achievement Level _________________________

Part Two

1. Do you understand what an AI chatbot is? [Single choice question] *

○Not at all

○Not really

○Somewhat

○Quite well

○Very well

2. Do you agree with the use of AI chatbots for study or work? [Single choice question] *

○Strongly disagree

○Disagree

○Neutral

○Agree

○Strongly agree

3. Have you used an AI chatbot in your study process? [Single choice question] *

○Never used (please jump to question 16)

○Occasionally used, please list at least one chatbot product you have used and whether you used it on its official website _________________ * (please jump to question 14)

○Frequently used, please list at least one chatbot product you have used and whether you used it on its official website _________________ * (please jump to question 14)

4. Does the current AI chatbot usage process align with your native language habits? [Single choice question] *

○Yes

○No

5. How often do you use this AI chatbot? [Single choice question] *

○Daily

○Several times a week

○About once a week

○Irregularly, based on need

○Rarely, only in specific situations

6. If you have not used it before, are you willing to learn to use an AI chatbot? [Single choice question] *

○Absolutely not (please jump to question 17)

○Not willing (please jump to question 17)

○Neutral (please jump to question 18)

○Somewhat willing (please jump to question 18)

○Very willing (please jump to question 18)

Depends on the first option of question 3

7. If you are not willing to use AI chatbots, what is the main reason? [Multiple choice question] *

□No need

□No interest

□Inconvenient to use

□Concerned about privacy issues

□Worried about inaccurate information provided

□Other (please specify) _________________*

Depends on the first and second options of question 6

8. If a high-quality, convenient AI chatbot is provided to assist your study, how much are you willing to pay per month to use it? [Single choice question] *

○Free

○Less than 20 yuan

○20-50 yuan

○50-100 yuan

○More than 100 yuan

Depends on the third, fourth and fifth options of question 6 and the second and third options of question 3

9. What is your primary purpose for using an AI chatbot? Please select the answer that best fits your purpose from the options below, or you can choose "Other" and fill in your own purpose. [Ranking question, please fill in the numbers in order in the brackets] *

[ ] Quickly obtain basic medical information and knowledge (e.g.: quick access to medical knowledge about diseases, symptoms, treatment plans, etc.)

[ ] Seek answers and guidance for complex medical problems (e.g.: solve medical problems encountered in learning or clinical practice, get guidance and suggestions.)

[ ] Self-health management and self-diagnosis (e.g.: obtain health management advice, self-diagnosis tools or guidance to improve your own health status).

[ ] Explore new research and academic resources (e.g.: access the latest research literature, academic resources or participate in academic discussions to expand your own medical knowledge and research horizons).

[ ] Improve medical learning and training experience (e.g.: enhance the experience of medical learning and training, deepen understanding and consolidate knowledge through interaction and dialogue with robots).

[ ] Improve learning efficiency (e.g. speed up literature retrieval, assist in writing article abstracts, assist in literature translation, etc.)

[ ] As with general search engines, retrieve all kinds of information

[ ] Chatting, entertainment

[ ] Other (please fill in your own purpose)

10. What advantages do you think AI chatbots have? (Ranking question, please fill in the numbers in order in the brackets)

Please rank the importance starting from 1 [Ranking question, please fill in the numbers in order in the brackets] *

[ ] They can provide quick, accurate medical information and diagnostic results

[ ] They can effectively help medical students learn and master medical knowledge

[ ] They can alleviate the work pressure and burden of doctors

[ ] They can improve the efficiency and quality of medical services

[ ] Other (please specify)

11. What disadvantages or risks do you think AI chatbots have? (Ranking question, please fill in the numbers in order in the brackets)

Please rank the importance starting from 1 [Ranking question, please fill in the numbers in order in the brackets] *

[ ] There may be a risk of data privacy leakage

[ ] It may lead to the degradation or unemployment of doctor skills

[ ] There may be a risk of misdiagnosis or missed diagnosis

[ ] It may reduce the human touch and humanization of medical services

[ ] Other (please specify)

Part III

If you have used an AI chatbot, please answer the following questions based on your actual experience of using an AI chatbot. If you haven't used an AI chatbot, you can also answer according to your true thoughts.

1. Please use "strongly disagree", "disagree", "neutral", "agree", and "strongly agree" to answer the following questions. [Matrix single choice question] *

|  | Strongly Disagree | Disagree | Neutral | Agree | Strongly Agree |
| --- | --- | --- | --- | --- | --- |
| 1.AI chatbots are (will be) very useful for my learning. | ○ | ○ | ○ | ○ | ○ |
| 2.Using AI chatbots will (can) allow me to complete learning or work tasks faster. | ○ | ○ | ○ | ○ | ○ |
| 3.Using AI chatbots can (will) improve my learning or work efficiency. | ○ | ○ | ○ | ○ | ○ |
| 4.Using AI chatbots can (will) improve my academic performance or work performance. | ○ | ○ | ○ | ○ | ○ |
| 5. I find the AI chatbot interface clear, and its usage and operation process are easy to understand. | ○ | ○ | ○ | ○ | ○ |
| 6. I believe I can use AI chatbots proficiently. | ○ | ○ | ○ | ○ | ○ |
| 7. I believe using AI chatbots is easy. | ○ | ○ | ○ | ○ | ○ |
| 8. I believe learning how to operate AI chatbots is easy for me. | ○ | ○ | ○ | ○ | ○ |
| 9.People who influence my behavior (such as classmates, colleagues, and friends) think I should use AI chatbots. | ○ | ○ | ○ | ○ | ○ |
| 10.People who are important to me (such as department heads, mentors, superiors, and hospital leaders) think I should use AI chatbots. | ○ | ○ | ○ | ○ | ○ |
| 11.The health administration department provides help in using AI chatbots. | ○ | ○ | ○ | ○ | ○ |
| 12.Overall, my school, hospital, and department all support the use of AI chatbots. | ○ | ○ | ○ | ○ | ○ |
| 13.I have the resources needed to use AI chatbots (such as equipment, support from classmates or colleagues, information networks, etc.) | ○ | ○ | ○ | ○ | ○ |
| 14. I have the knowledge required to use AI chatbots. | ○ | ○ | ○ | ○ | ○ |
| 15.AI chatbots are incompatible with other systems I use. | ○ | ○ | ○ | ○ | ○ |
| 16.When I encounter difficulties in using AI chatbots, there will be professionals to help. | ○ | ○ | ○ | ○ | ○ |
| 17.I intend to use AI chatbots in the next two months. | ○ | ○ | ○ | ○ | ○ |
| 18.I plan to use AI chatbots in the next two months. | ○ | ○ | ○ | ○ | ○ |
| 19. I have confirmed that I will use AI chatbots in the next two months. | ○ | ○ | ○ | ○ | ○ |
| 20.I may need more time to correct errors of AI chatbots. | ○ | ○ | ○ | ○ | ○ |
| 21.I think AI chatbots may have performance failures or immature technologies that cannot provide accurate information and mislead my learning or work. | ○ | ○ | ○ | ○ | ○ |
| 22. I worry that AI chatbots will leak my private information. | ○ | ○ | ○ | ○ | ○ |
| 23. I don't want AI chatbots to change my learning or work style, because these new AI tools are unfamiliar to me. | ○ | ○ | ○ | ○ | ○ |
| 24. I don't want to use AI chatbots because past experiences tell me that these new high-tech products always perform poorly in practical applications. | ○ | ○ | ○ | ○ | ○ |
| 25.I don't want to use AI chatbots for fear of losing my job, because AI-assisted technology might do better than me. | ○ | ○ | ○ | ○ | ○ |
| 26.If I hear about a new information technology, I will find ways to try it. | ○ | ○ | ○ | ○ | ○ |
| 27.Among my peers, I am usually the first to try new information technology. | ○ | ○ | ○ | ○ | ○ |
| 28.Overall, I am hesitant to try new information technologies. | ○ | ○ | ○ | ○ | ○ |
| 29. I like to experience new information technologies. | ○ | ○ | ○ | ○ | ○ |
| 30.I frequently use AI chatbots. | ○ | ○ | ○ | ○ | ○ |
| 31.I have recommended using AI chatbots to others. | ○ | ○ | ○ | ○ | ○ |
| 32.I will continue to use AI chatbots. | ○ | ○ | ○ | ○ | ○ |
| 33.I believe there should be better AI chatbots to replace the one I am currently using. | ○ | ○ | ○ | ○ | ○ |

If you are interested in learning more about the progress of this research and receiving a gift, you can leave your email address here for notifications. Your email: [Fill in the blank]

_________________________________

Thank you for participating in this survey!
